# Effect of in ovo feeding of xylobiose and xylotriose on plasma immunoglobulin, cecal metabolites production, microbial ecology, and metabolic pathways in broiler chickens

**DOI:** 10.1186/s40104-024-01022-7

**Published:** 2024-05-04

**Authors:** Razib Das, Pravin Mishra, Birendra Mishra, Rajesh Jha

**Affiliations:** Department of Human Nutrition, Food and Animal Sciences, College of Tropical Agriculture and Human Resources, University of Hawaii at Manoa, Honolulu, HI 96822 USA

**Keywords:** Broiler, In ovo, Metagenomics, Prebiotic, Xylooligosaccharides

## Abstract

**Background:**

Dietary supplementation of xylooligosaccharides (XOS) has been found to influence gut health by manipulating cecal microbiota and producing microbe-origin metabolites. But no study investigated and compared the effect of in ovo feeding of xylobiose (XOS2) and xylotriose (XOS3) in chickens. This study investigated the effect of in ovo feeding of these XOS compounds on post-hatch gut health parameters in chickens. A total of 144 fertilized chicken eggs were divided into three groups: a) non-injected control (CON), b) XOS2, and c) XOS3. On the 17^th^ embryonic day, the eggs of the XOS2 and XOS3 groups were injected with 3 mg of XOS2 and XOS3 diluted in 0.5 mL of 0.85% normal saline through the amniotic sac. After hatching, the chicks were raised for 21 d. Blood was collected on d 14 to measure plasma immunoglobulin. Cecal digesta were collected for measuring short-chain fatty acids (SCFA) on d 14 and 21, and for microbial ecology and microbial metabolic pathway analyses on d 7 and 21.

**Results:**

The results were considered significantly different at *P* < 0.05. ELISA quantified plasma IgA and IgG on d 14 chickens, revealing no differences among the treatments. Gas chromatography results showed no significant differences in the concentrations of cecal SCFAs on d 14 but significant differences on d 21. However, the SCFA concentrations were lower in the XOS3 than in the CON group on d 21. The cecal metagenomics data showed that the abundance of the family Clostridiaceae significantly decreased on d 7, and the abundance of the family Oscillospiraceae increased on d 21 in the XOS2 compared to the CON. There was a reduction in the relative abundance of genus *Clostridium sensu stricto *1 in the XOS2 compared to the CON on d 7 and the genus *Ruminococcus torques* in both XOS2 and XOS3 groups compared to the CON on d 21. The XOS2 and XOS3 groups reduced the genes for chondroitin sulfate degradation I and L-histidine degradation I pathways, which contribute to improved gut health, respectively, in the microbiome on d 7. In contrast, on d 21, the XOS2 and XOS3 groups enriched the thiamin salvage II, L-isoleucine biosynthesis IV, and O-antigen building blocks biosynthesis (*E. coli*) pathways, which are indicative of improved gut health. Unlike the XOS3 and CON, the microbiome enriched the pathways associated with energy enhancement, including flavin biosynthesis I, sucrose degradation III, and Calvin-Benson-Bassham cycle pathways, in the XOS2 group on d 21.

**Conclusion:**

In ovo XOS2 and XOS3 feeding promoted beneficial bacterial growth and reduced harmful bacteria at the family and genus levels. The metagenomic-based microbial metabolic pathway profiling predicted a favorable change in the availability of cecal metabolites in the XOS2 and XOS3 groups. The modulation of microbiota and metabolic pathways suggests that in ovo XOS2 and XOS3 feeding improved gut health during the post-hatch period of broilers.

## Introduction

During egg formation, the chicken egg acquires microbiota through vertical transmission from the maternal oviduct [1]. The dynamic microbiota present within a chicken egg plays a vital role in its embryonic development. The early gut health, gut microbiota, nutrient utilization, and immune status of chickens significantly influence broiler growth and development [2, 3]. During the post-hatch life, dietary ingredients can influence health and growth performance by modifying gut microbiota and metabolite production in the intestine [4]. The modification of gut microbiota in embryos has the potential for inducing changes at an early stage of development, prompting significant interest among researchers [3]. A comparison of the microbial compositions of chicken embryos among three developmental stages (early, middle, and late stages) showed that the embryos on d 19, a late stage of development, harbored more diverse microbiota than the embryos on d 3 or d 12 [5]. So, an intervention during the late stage of embryogenesis has a higher potential to influence the microbial diversity of the embryos.

Prebiotics are effective methods for modifying the gut microbiota to improve overall health. Though the definition of prebiotics evolved at various times, the International Scientific Association for Probiotics and Prebiotics updated the definition of prebiotics in 2016 as: “a substrate that is selectively utilized by host microorganisms conferring a health benefit” [6]. This definition is not limited to the function of the prebiotics as a selective growth promoter of certain bacterial genera but is directed to finding their potential to increase gut microbial diversity. Studies have shown that injecting prebiotics in ovo can effectively supplement nutrients to the embryo [4]. Several in ovo studies examined the effects of prebiotics in post-hatch chickens and reported ample advantages emanating from in ovo injection of inulin [7], galactooligosaccharides [8], raffinose-family oligosaccharides [9], xylooligosaccharides (XOS) and mannanoligosaccharides [10], etc.

Xylooligosaccharides are produced from the lignocellulosic contents of the plants through hydrolysis reaction [11]. The xylobiose and xylotriose are the oligomers within XOS group, which contain two and three xylose units, respectively, in their structures. Different studies showed that regular dietary supplementation of XOS can effectively feed the gut microbiome and change the gut metabolite composition [12, 13], but the effects of in ovo feeding of XOS are yet to be thoroughly understood. Other studies found that in ovo stimulation with bioactive compounds can stimulate the gut microbiota before hatch and lead to regulating cecal microbiota, short-chain fatty acids (SCFA) production, and immunity in adult broilers [10, 14, 15]. However, there is a scarcity of information regarding the effect of in ovo XOS feeding on inducing changes in post-hatch broiler chickens.

The objective of this study was to evaluate the effect of in ovo XOS feeding on broiler chickens’ plasma immunoglobulin, cecal metabolites production, microbial ecology, and MMPs during post-hatch days. We hypothesized that the in ovo XOS feeding could alter the concentration of plasma immunoglobulin, cecal microbiota, and availability of cecal metabolites in broiler chickens.

## Materials and methods

### In ovo feeding

A total of 144 fertilized eggs from Cobb 500 hens were obtained from Asagi Hatchery Inc. (Honolulu, HI, USA) on the 15^th^ day of embryonic development. Then, the eggs were carefully transferred to incubators set at 37.5 °C and a relative humidity of 58%. The eggs were then divided into three treatment groups based on the type of in ovo feeding they would receive: XOS2, XOS3, or no in ovo feeding (CON). The XOS2 (purity > 95%) and XOS3 (purity > 90%) were sourced from Megazyme International Ireland Ltd., Bray, Ireland. The solution for the in ovo feeding was prepared at a concentration of 6 mg of XOS2 or XOS3 in each mL of 0.85% normal saline, following the same methodology of a previous study that investigated the effects of in ovo xylotriose and xylotetraose [10]. Our study did not include a vehicle control group, such as a saline-injected group. Previous studies demonstrated no significant changes between the in ovo saline feeding and non-injection control groups [10, 16, 17]. Considering the result of these studies, we excluded the saline group from the current study. As the in ovo feeding at the later stage of embryonic development could affect the gut microbiota development [5], in ovo XOS feeding of embryos was carried out on the 17^th^ embryonic day in a biosafety cabinet as previously described [10]. The broad end of eggs was chosen for the injection of XOS solution and was disinfected with 10% povidone iodine prior to stabbing with a disinfected awl. Then, 0.5 mL of XOS solution that contained 3 mg of XOS was injected using blunt tip 21 gauze sterile needles to the amniotic sac of the embryos, and the process ended by attaching a piece of parafilm over the site of injection.

### Chick management and sample collection

A total of 131 eggs hatched, and the chicks from each treatment group were distributed to six replicate pens (no less than five chicks/pen). The chicks were raised according to the Cobb 500 guidelines and had ad libitum access to feed and water. The chickens were fed a corn-soybean meal-based starter mash diet throughout the experimental period, which was formulated to meet or surpass the nutritional requirements of broiler chickens [18].

On each sample collection day (d 7, 14, and 21), one chicken from each pen was euthanized using CO_2_ gas, resulting in six chickens from each treatment group. Blood samples were collected (on d 14) using vacutainers coated with K_2_EDTA to measure plasma immunoglobulin concentration. Subsequently, the vacutainers underwent centrifugation to separate the plasma from the blood, which was then stored at –20 °C until the ELISA (enzyme-linked immunosorbent assay) was conducted. Furthermore, cecal digesta were collected for SCFA (on d 14 and 21) and metagenomic analysis (on d 7 and 21). The cecal digesta were preserved in the refrigerator following the process described by Singh et al. [19] until analysis.

### Plasma immunoglobulins

Following the manufacturer’s instructions, the commercial ELISA kit (Bethyl Laboratories, Montgomery, TX, USA) was used to measure the chicken plasma immunoglobulins, IgA and IgG. Ten standard gradients for IgA (1,000, 333, 111, 37.04, 12.35, 4.12, 1.37, 0.456, 0.152, and 0 ng/mL) and 12 standard gradients for IgG (500, 166.67, 55.56, 18.52, 6.17, 2.06, 0.69, 0.23, 0.08, 0.03, 0.009, and 0 ng/mL) were created following serial dilutions. The plasma samples were diluted by a factor of 1:1,000 and 1:100,000, respectively, using a dilution buffer that came with the kit for IgA and IgG analyses. Then 100 µL of each standard solution or diluted plasma, each in duplicates, was pipetted and transferred to the wells of the ELISA plate. After 1 h of room temperature incubation, the plate was washed. Later, 100 µL of detection antibody was added to the well, followed by 1 h of room temperature incubation, and washed at the end. The colorimetric reaction was initiated by adding streptavidin-conjugated horseradish peroxidase and TMB substrate, and the mixture was left to be catalyzed for 30 min in the dark. The Stop Solution was added to terminate the reaction and the absorbance at 450 nm was measured immediately using an ELISA plate reader (SynergyLX, Biotek, Santa Clara, CA, USA).

### Cecal SCFA analysis

After thawing the cecal digesta, approximately 200 mg of digesta was processed to measure the concentrations of short-chain fatty acids (acetate, butyrate, and propionate). A mixture of deionized water, trimethyl acetic acid, and metaphosphoric acid was added to the digesta. The volume of each component in the mixture was determined as previously described [19]. The resulting solution was then subjected to centrifugation, and the supernatant was collected in vials and analyzed using gas chromatography (GC) following the method described by Singh et al. [19]. The GC was performed using a Trace 1300 gas chromatograph (Thermo Scientific, Waltham, MA, USA) equipped with an automated AS 1310 injector (Thermo Scientific) and a flame ionization detector. The external standard preparation and instrument setup were conducted following the procedure outlined by Singh et al. [19]. The GC signals were analyzed using the Chromeleon 7.2 software (Thermo Scientific) to determine the concentration of SCFA.

### Microbial DNA extraction, 16S rRNA amplicon sequencing, and metagenomic analysis

Microbial DNA was extracted from the microbes of cecal digesta using QIAamp^®^ fast DNA stool mini kit (Qiagen, Catalog Number: 51604), following the protocol recommended by the manufacturer. The DNA concentration was measured using a UV–visible spectrophotometer (NanoDrop™ One, Thermo Scientific, Madison, WI, USA), and the DNA samples were sent to the Advanced Studies in Genomics, Proteomics and Bioinformatics facility of UH Manoa for Illumina MiSeq Sequencing.

Sequencing and amplification of hypervariable V3 and V4 regions of the 16S rRNA was carried out for the preparation of 16S rRNA library. The method of library preparation, including selection of the primers for amplicon PCR, specifications of the adapters, PCR protocol, was similar to the previously described procedure [19]. After normalization and pooling of the amplicons, the amplicons were sequenced using the Illumina MiSeq benchtop sequencer.

### Metagenomic sequencing and data analysis

Paired-end sequences generated by MiSeq Sequencer were processed using QIIME2-2021.4 [20]. The paired-end sequences were demultiplexed and quality filtered using the demux plugin, following sequence quality control and feature table construction using the DADA2 plugin [21]. The q2-feature-classifier plugin for QIIME2 [22] and the classify-sklearn naive Bayes taxonomy classifier [23] pre-trained on the Greengenes 13_8 99% OTUs reference sequences [24] were used to assign taxonomic classification to ASVs. Feature table was exported into a tab-separated values file in order to use in R studio [25]. Samples from d 7 and 21 were rarefied to 23,984 and 32,584 sequences per sample, respectively, through repeatable random subsampling without replacement using the phyloseq package for R [26]. Alpha diversity indices (Observed, Chao1, Shannon, Simpson), beta diversity indices, and relative abundances of bacteria at different taxa were calculated and plotted using the phyloseq and ggplot2 in R [26, 27].

### Statistical analyses

The individual chickens from pens were considered as the experimental unit in this study. The plasma immunoglobulins and cecal SCFA concentrations were analyzed using one-way ANOVA in R [28] and R studio [25]. The differences among the treatments were considered significant at a *P* < 0.05. The calculated significant differences among the treatments were separated by Tukey’s HSD test. As the cecal microbiome data were non-parametric, a Kruskal–Wallis test was applied to analyze the alpha diversity, and a permutational multivariate analysis of variance was applied for the beta diversity. The group-to-group comparison of the cecal microbial abundance and MMPs was analyzed in STAMP [29], which followed a two-sided White’s non-parametric *t*-test with DP:bootstrap at 0.95.

## Results

### Plasma immunoglobulins

**Fig. 1:**
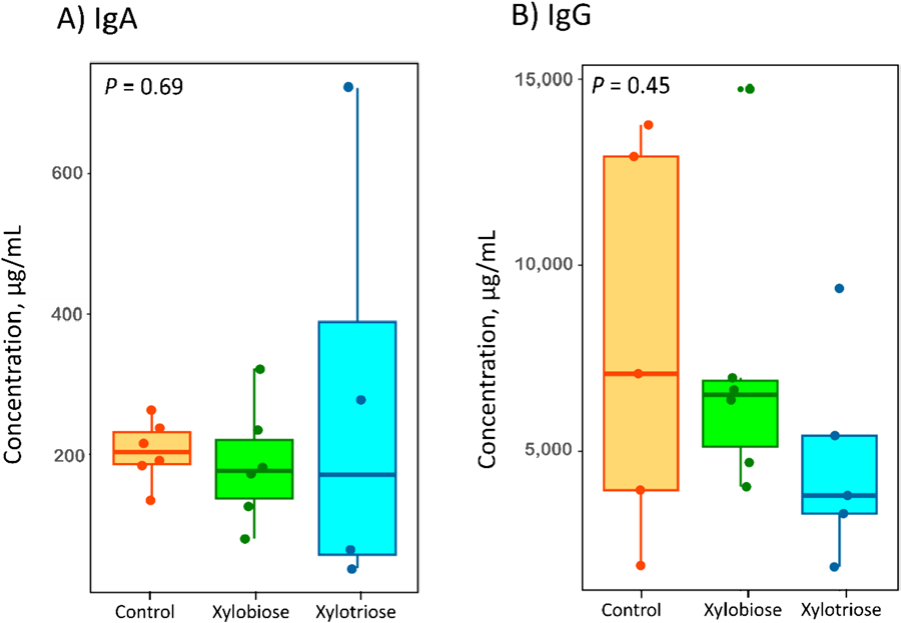
Effects of in ovo XOS feeding on plasma immunoglobulin (Ig) concentration on d 14. **A** concentration of IgA; **B** concentration of IgG. The number of samples per treatment group was ≥ 4 to 6. The *P* values are shown in the top left corner of the plots. The level of significance was considered at *P* < 0*.*05

The in ovo feeding of XOS did not significantly influence the concentration of plasma IgA (*P* = 0.689) and IgG (*P* = 0.454) in the chickens on d 14 (Fig. 1).

### Cecal short chain fatty acids

**Fig. 2:**
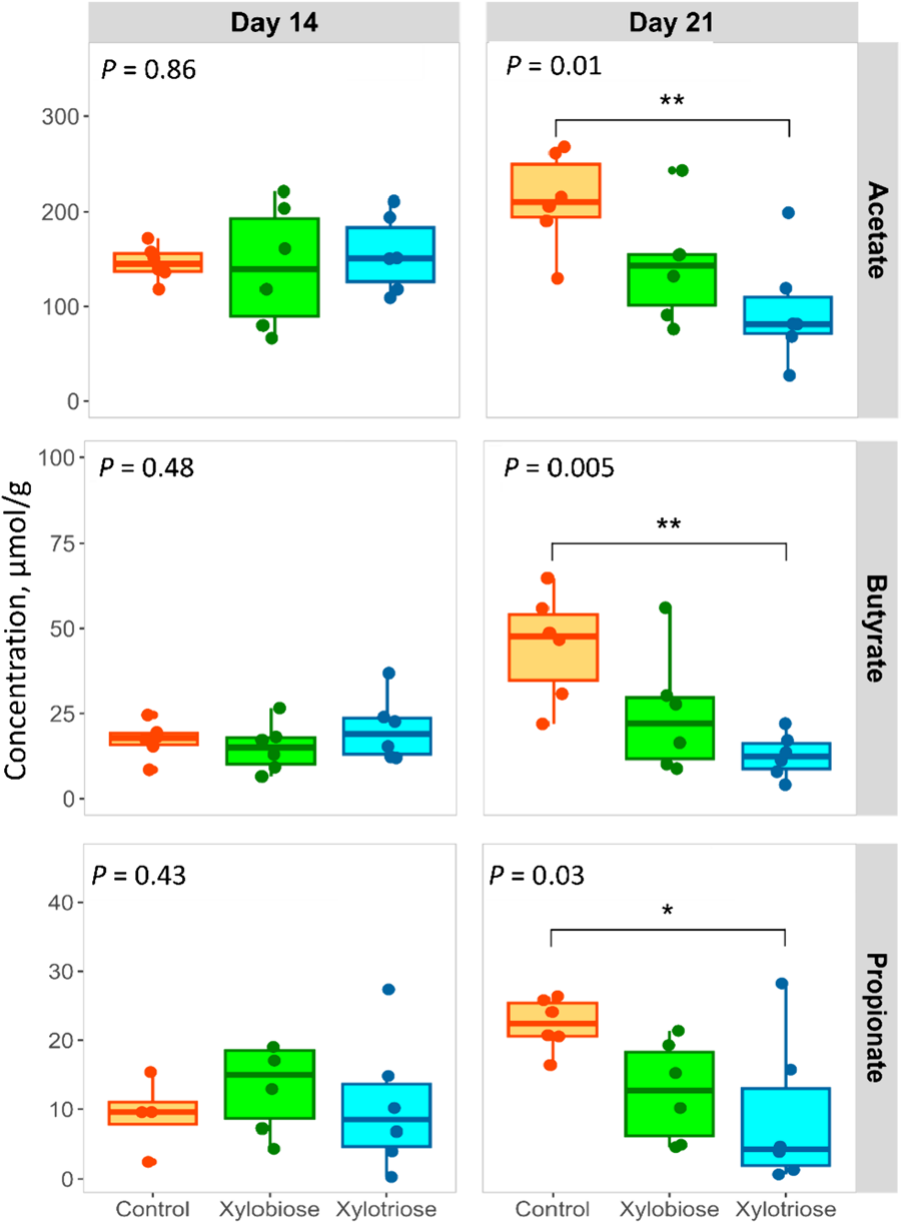
Effects of in ovo XOS feeding on the concentration of short-chain fatty acids in the cecal digesta on d 14 and 21. The level of significance was considered at *P* < 0.05. The *P* values are shown in the top left corner of each plot. Asterisks indicate significant differences between treatment groups, determined by Tukey’s multiple comparisons of means after one-way ANOVA (^*^P < 0.05; ^**^P < 0.01; ^***^P < 0.001)

The concentrations of three types of SCFA were measured on d 14 and d 21. The analysis of d 14 cecal digesta revealed no significant difference in the concentration of these SCFA among the three treatments (Fig. 2). On d 21, the XOS3 group significantly lowered the SCFA concentrations compared to the CON group (Fig. 2).

### Cecal microbiota composition

**Fig. 3:**
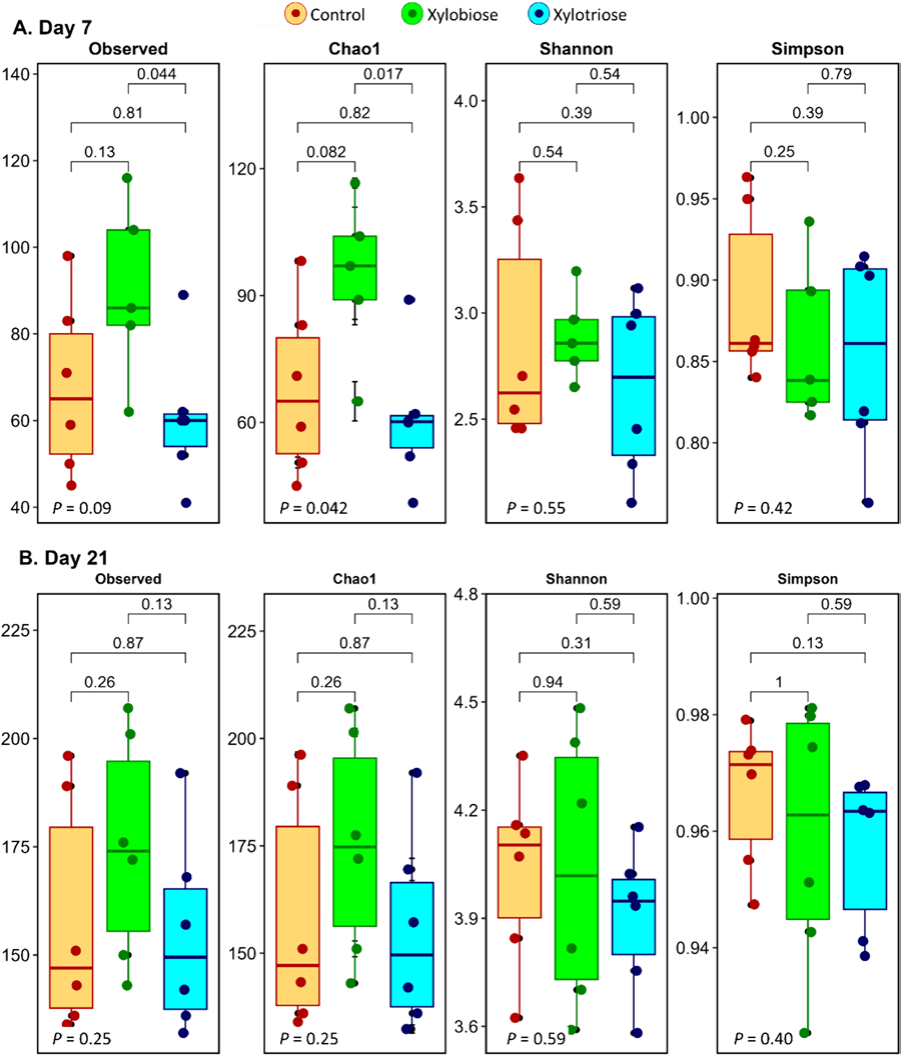
Effects of in ovo XOS feeding on alpha diversity indices of cecal microbiota on: **A** d 7, **B** d 21. The level of significance was considered at *P* < 0.05. A Kruskal–Wallis test was performed to compare the means of the groups and for pairwise comparisons. The global *P* values of tests are shown at the bottom left corner of each plot, and the *P* values for the pairwise comparisons are shown above the bars connecting the two groups

**Fig. 4:**
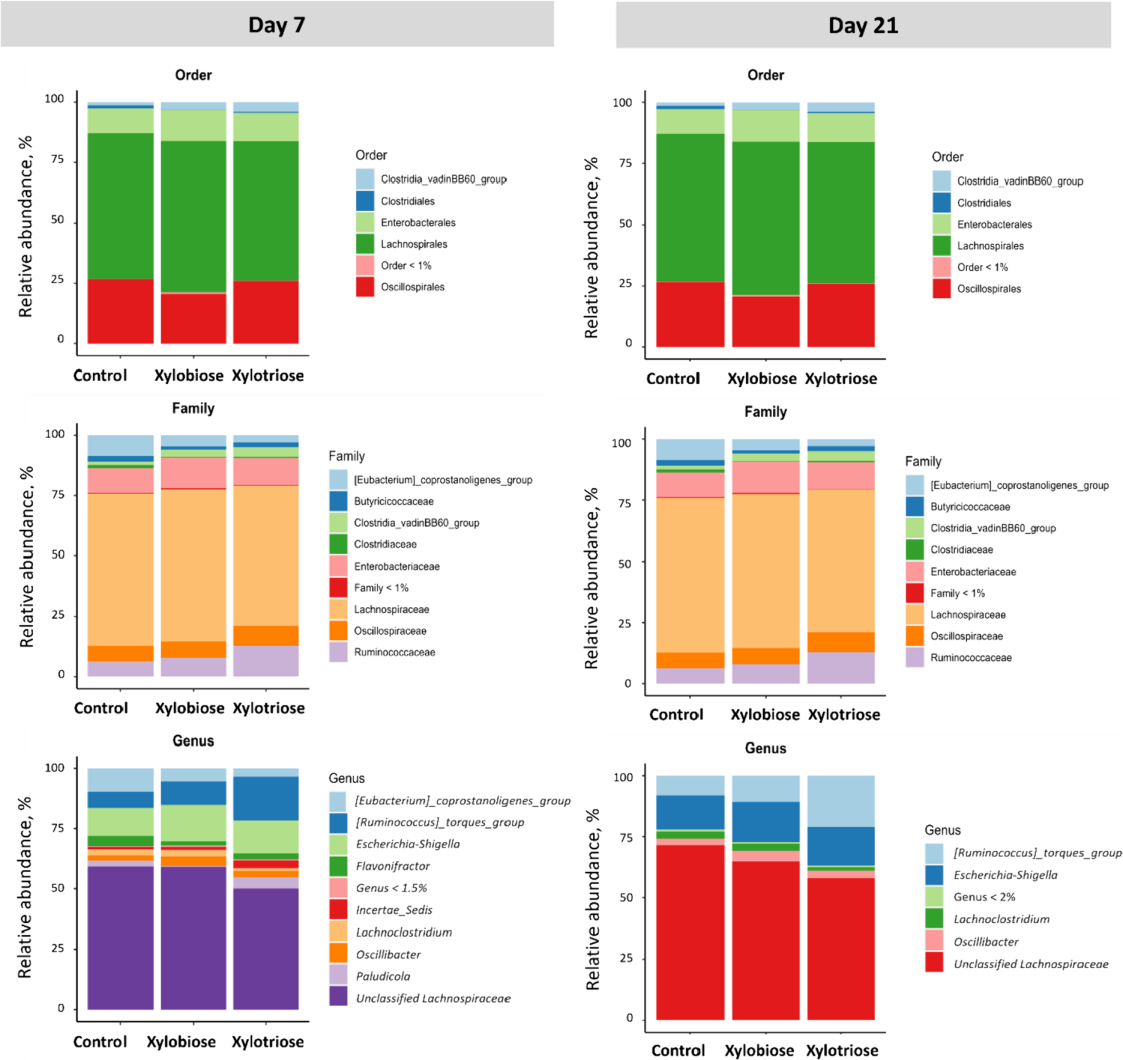
Effects of in ovo XOS feeding on the relative abundance of most frequent bacteria in cecal digesta on d 7 and 21 at the order (top row), family (middle row), and genus (bottom row) levels of taxonomic classification

**Fig. 5:**
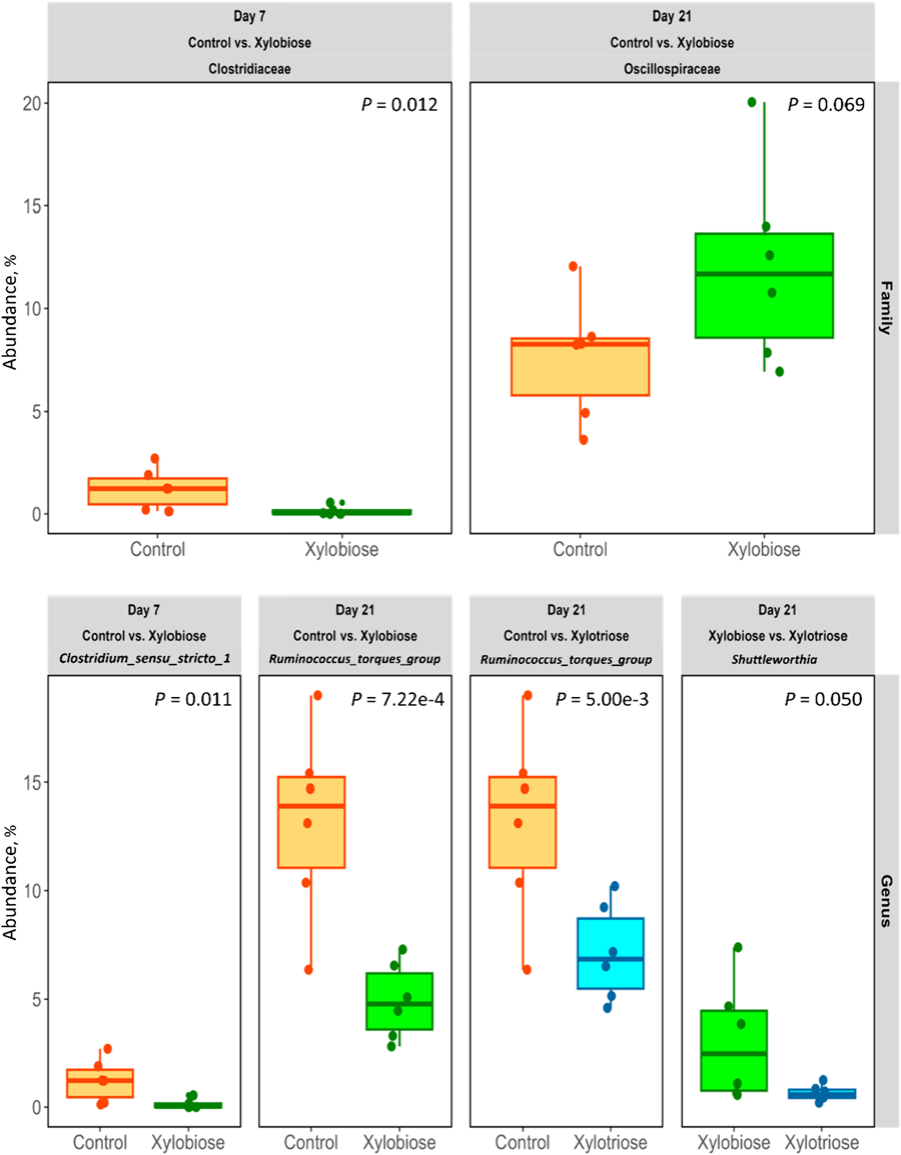
The effects of in ovo XOS on the relative abundance of bacteria at the family (top row) and genus (bottom row) levels of taxonomic classification of bacteria. The texts in column facets include information on the day, pair of treatments, and bacterial taxonomic classification. The texts in row facets represent the levels of the taxa. White’s non-parametric two-sided *t*-test was performed in STAMP v2 software with DP:bootstrap at 0.95 CI. The level of significance was considered at *P* < 0.05. The *P* values are shown in the top-right corner of each plot

The alpha diversity of the cecal microbiota measured on d 7 and 21 are presented in Fig. 3. In ovo XOS feeding efficiently changed the diversity of the microbiota that persisted till d 7, and the XOS2 group had a higher alpha diversity index than the XOS3 group (*P* = 0.044 for Observed, and *P* = 0.017 for Chao1 index) on d 7. Chao1 index showed a trend to increase in alpha diversity (*P* = 0.082) on d 7 in the XOS2 chickens compared to the CON chickens. With the aging of chickens, the alpha diversity among the treatments disappeared on d 21 (Fig. 3). The alpha diversity measured on the Shannon and Simpson indices showed no significant difference among the treatments on d 7 and 21. The relative abundance of cecal microbiota on d 7 and 21 are presented in Figs. 4 and 5.

**Fig. 6:**
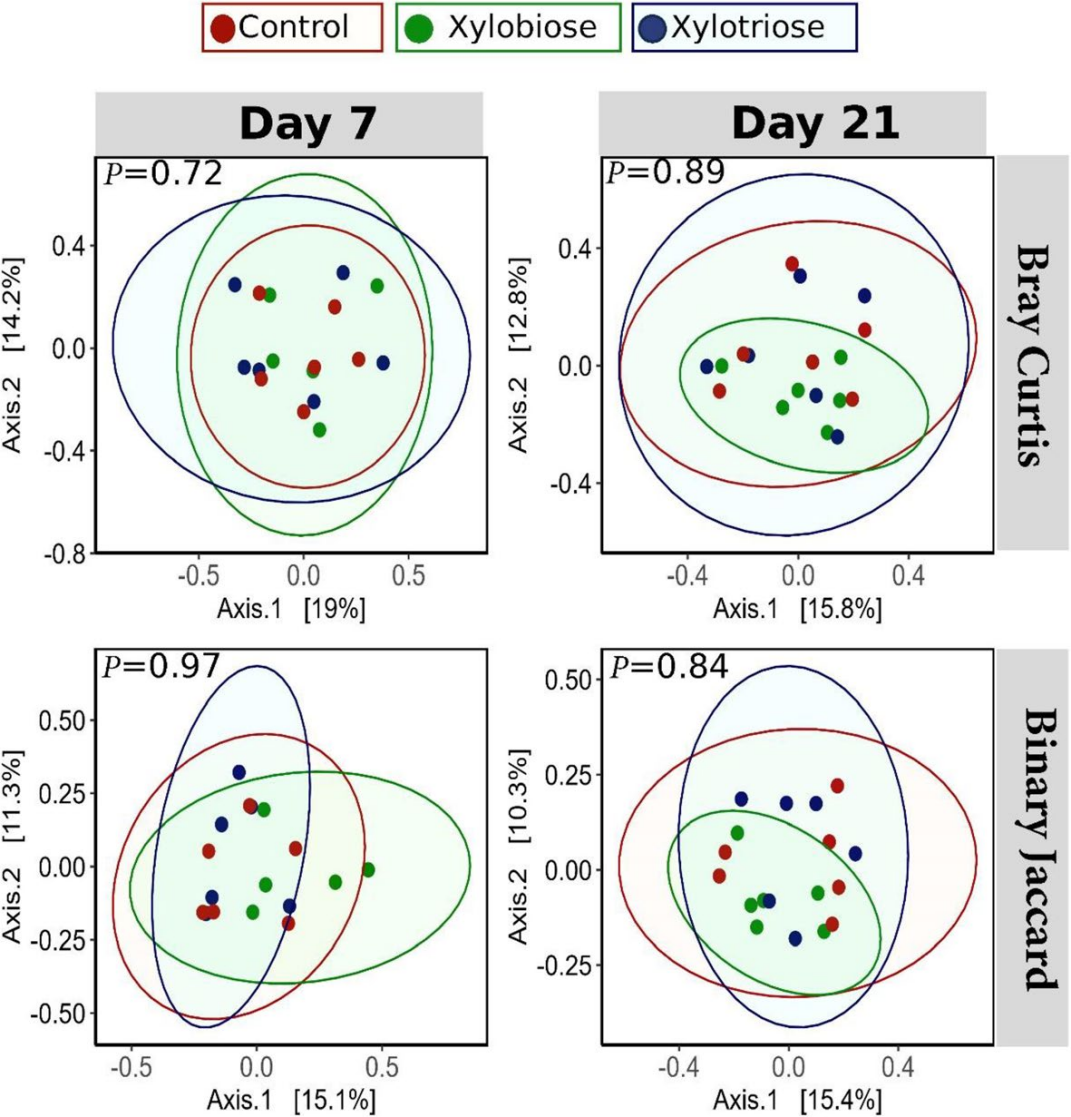
Principal coordinate analyses based on beta diversity indices. The rows represent the plots for Bray–Curtis (top) and Binary-Jaccard (bottom) indices. The columns represent the results from d 7 (left column) and d 21 (right column) cecal microbiota. The level of significance was considered at *P* < 0.05. The *P* values are on the top left corner of each plot. The study observed no significant differences in beta diversity among the treatments

Principal Component Analysis (PCA) was performed on the matrices of Jaccard distance and Bray–Curtis distance, which are, respectively, qualitative and quantitative beta diversity measures of the microbiota (Fig. 6). These beta-diversity analyses showed that the treatments had no difference in diversity in the cecal microbiota of d 7 and 21 chickens.

By conducting White’s non-parametric two-sided *t*-test on the abundance of bacteria at the family and genus levels of the taxonomic classification (Fig. 5), we found that the abundance of bacteria under the family Clostridiaceae (*P* = 0.012) and genus *Clostridium sensu stricto* 1 (*P* = 0.011) were decreased on d 7 in the XOS2 compared to the CON group. Whereas on d 21, the bacteria classified under the family Oscillospiraceae showed a trend to increase (*P* = 0.069) in abundance in the XOS2 compared to the CON. However, the genus *Ruminococcus torques* group decreased (*P* < 0.001) in the XOS2 (*P* = 7.22e^−4^) and XOS3 (*P* = 5.00e^−3^) compared to the CON group, and the genus *Negativibacillus* decreased (*P* = 0.039) in the XOS2 compared to the CON group.

### Microbial metabolic pathways

**Fig. 7:**
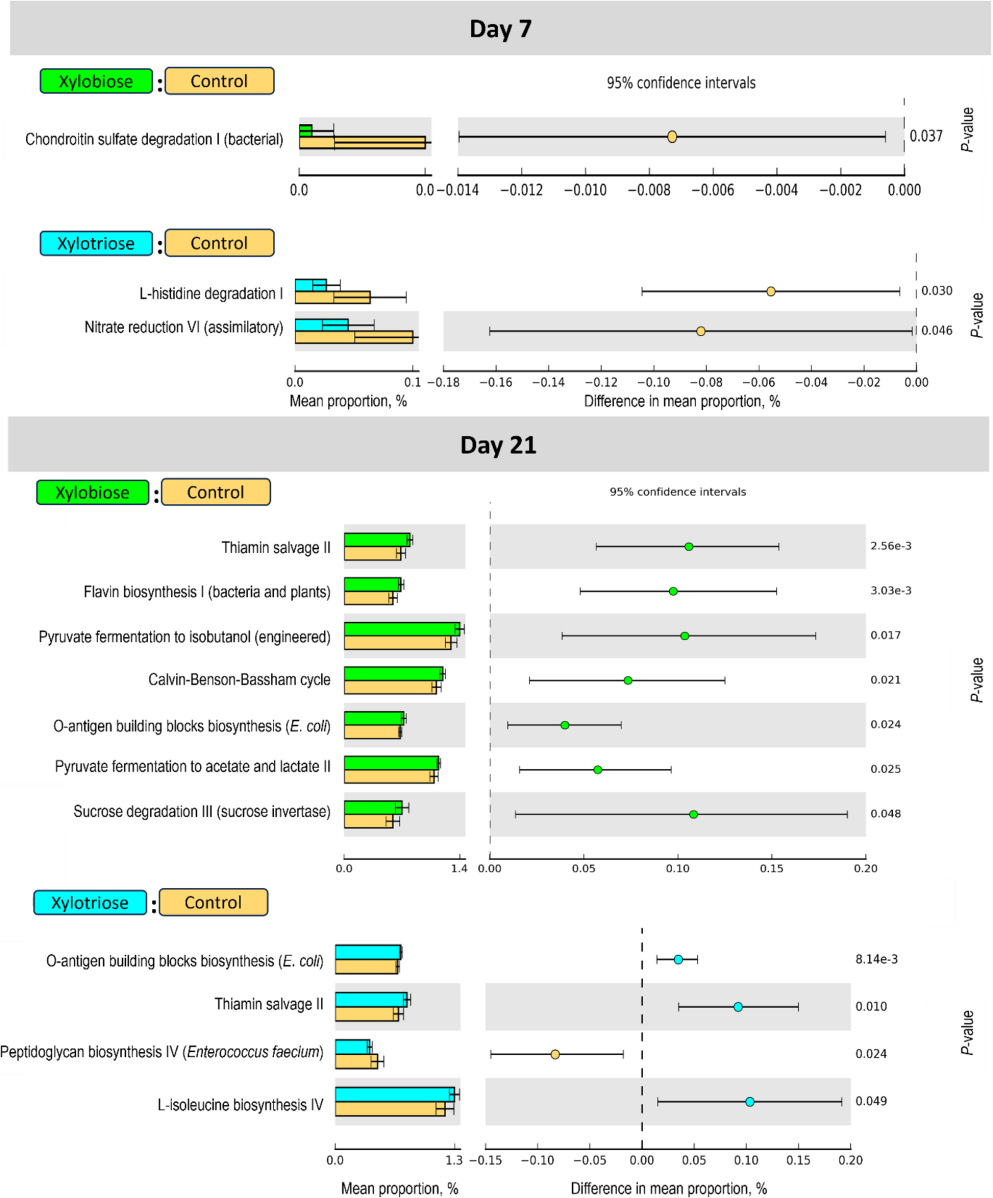
Effects of in ovo XOS on microbial metabolic pathways in the cecal digesta on d 7 and 21. The microbial metabolic pathways were compared between xylobiose and control, or between xylotriose and control. White’s non-parametric two-sided *t*-test was conducted in the statistical analysis of taxonomic and functional profile (STAMP v2) software with DP:bootstrap at 0.95 CI. The level of significance was considered at *P* < 0.05. The *P* values are shown at the rightmost side of each pathway

The XOS2 and XOS3 groups reduced the expression levels of genes for chondroitin sulfate degradation I (*P* = 0.037) and L-histidine degradation I (*P* = 0.030) pathways, respectively, in the microbiome on d 7 (Fig. 7). In contrast, these treatment groups increased the expression of genes involved in the thiamin salvage II (*P* < 0.001, when XOS2 was compared to CON; and *P* = 0.010, when XOS3 was compared to CON), L-isoleucine biosynthesis IV (*P* = 0.049, when XOS3 was compared to CON), and O-antigen building blocks biosynthesis (*E. coli*) (*P* = 0.024, when XOS2 was compared to CON; and *P* < 0.001, when XOS3 was compared to CON) pathways on d 21. Unlike the XOS3 and CON, the microbial gene annotation prediction showed an increase in flavin biosynthesis I (*P* < 0.01), sucrose degradation III (*P* = 0.048), and Calvin-Benson-Bassham cycle (*P* = 0.021) pathways in the XOS2 group on d 21.

## Discussion

Plasma immunoglobulin levels (such as, IgA and IgG) can fluctuate due to immunodeficiency, infections, or malignancies, and are indicative of humoral immune status. The impact of in ovo feeding of XOS on the modulation of plasma immunoglobulin (Ig) concentrations is not sufficiently researched. However, a study conducted by Ding et al. [30] has reported that dietary supplementation of XOS increased plasma IgA concentration in laying hens, whereas no significant effect was observed on plasma IgG concentration. Similarly, another study indicated that dietary chitooligosaccharides, a prebiotic, did not significantly change serum IgA and IgG concentrations in chickens on d 21 and d 42 [31]. The present study has examined the potential effect of in ovo feeding of XOS on the plasma IgA and IgG concentration. The results showed that on the 14^th^ d, there was no significant difference in the plasma IgA and IgG concentration due to in ovo XOS feeding. These findings suggest that in ovo feeding of XOS might not significantly alter the plasma immunoglobulin concentration in the post-hatch period. However, secretory IgA in intestinal mucosal secretions functions in a different way than the plasma immunoglobulins function and protects the microbial invasion and penetration of the intestinal mucosal lining [32]. Studies showed that in ovo feeding of prebiotics or synbiotics could potentially increase the secretory IgA concentration in broilers’ gut [16, 33]. We assume that measuring the secretory immunoglobulins in gut mucosal secretions would be beneficial to understand the mucosal defense due to in ovo XOS feeding.

The composition and diversity of microbiota in poultry’s gastrointestinal tract varies throughout the tract, from mouth to cloaca. Cecal microbial populations have been well studied in poultry, and it has been discovered that the microbiota responds differently toward various interventions. This gut microbiota reflects the health status of the hosts [34]. The diversity indices are used to get information on the complexity and relatedness of the gut microbiota among the treatments. Our study analyzed the Observed, Chao1, Shannon, and Simpon indices for alpha diversity. We did not detect any difference in the Observed index; however, we observed a significant variation in the Chao1 index among the treatments on d 7. According to the Observed and Chao1 indices, the XOS3 had lower diversity than the XOS2 group on d 7. Moreover, there was no significant difference for the Shannon and Simpson indices on d 7 and the Observed, Chao1, Shannon and Simpson indices for alpha diversity on d 21. Similarly, the beta diversity indices showed that the treatment had no significant impact on cecal microbial diversity. Interestingly, the interpretation of diversity indices is arbitrary, and assuming higher diversity as inherently advantageous is not warranted [35]. Perhaps the abundance of certain bacterial species has a greater impact on the overall health of the host than the diversity because of the presence of many non-significant species in the ceca. So, we analyzed whether the treatment brought any significant changes in the abundance of bacteria for certain taxa.

We found that on d 7 the XOS2 significantly reduced the abundance of the Clostridiaceae family, which is a dominant family in the young chickens [36]. A study by Terada et al. [37] observed that a modified gut microbiota containing a smaller population of Clostridiaceae family increased the expression of proinflammatory and anti-inflammatory cytokines in the chickens. It is suggestive that in ovo feeding of XOS2 could benefit the chickens during the first week because of the plausible positive immune response derived from the reduction of the abundance of Clostridiaceae family. But, this relationship needs to be tested and understood with further research.

A higher abundance of *Clostridium sensu stricto* 1 in the gut indicates compromised gut barrier functionality [38]. Chen et al. [39] observed that XOS-diet could significantly decrease the abundance of *Clostridium sensu stricto* 1 in the ileum and colon of weaning piglets. Similarly, this study also found that the XOS2 significantly reduced *Clostridium sensu stricto* 1 in the ceca on d 7. Possibly, a decrease in the abundance of this genus on d 7 could improve the mucosal barrier function. The mucosa seemed to perform better till d 21 and increase the utilization of SCFAs, which led to a reduced concentration of SCFAs in the cecum on d 21.

Stanley et al. [40] showed an association between the increase of *Clostridium*, under the Family Clostridiaceae, and poor broiler performance. On d 7, the XOS2 group showed a significant decrease in the abundance of *Clostridium sensu stricto* 1. A study by Yang et al. [41] showed that the jejunal increase of *Clostridium sensu stricto* 1 occurs along with the decrement of *Lactobacillus *genus*,* where the abundance of *Clostridium sensu stricto* 1 leads to the progression of necrotic enteritis in the *Clostridium perfringens* challenged chickens*.* The significant reduction of this genus occurred in the XOS2 group, which could help reduce the proliferation of *Clostridium perfringens* and *Eimeria*, and contribute to preventing necrotic enteritis during the first week.

On d 21, the lower abundance of *Ruminococcus torques* in the XOS2 and XOS3 coincided with the lower production of SCFAs. This bacterium is known for fermenting fiber and produces SCFAs. The lower abundance of this species and lower concentration of SCFAs in the colon suggest a lack of fiber availability in the cecum. The reason could be the increased digestion of fibers in the distal ileum or the competitive exclusion of *Ruminococcus* from the cecum. On d 21, the abundance of *Ruminococcus torques* significantly increased in the CON, compared to the XOS2 and XOS3. A study by Wang et al. [38] reported that their abundance led to IFN-g upregulation, which could trigger cell-mediated immunity [42]. In addition, these bacteria produce glycosidases [43] and act as mucolytic bacteria [44]. Their abundance can impair the gut barrier function in the intestine. The reduction of its abundance in two XOS groups means that their ceca might have developed better gut integrity than the ceca of the control group.

Dietary supplementation of XOS for 21 d significantly reduced the abundance of *Negativibacillus* in a study by Wang et al. [45]. This genus often flourishes when the hosts go through a condition, e.g., gut dysbiosis, obesity-related disorders, colitis, etc. [46, 47]. There was a significant decrease in the abundance of the *Negativibacillus* genus in the XOS2 group compared to the CON group on d 21. This indicates better gut health in the XOS2 group.

The in ovo feeding of XOS2 and XOS3 played a significant role in modifying the complex cecal microbiota. We observed a reduction in the abundance of the family Clostridiaceae and genus *Clostridium sensu stricto* 1 in the XOS2 group compared to the CON group on d 7. This modification was followed by a reduction in the population of *Ruminococcus torques* group on d 21 in the XOS2 and XOS3 compared to the CON. Our findings implied a gut microbiota modification on d 7 and 21 had resulted due to in ovo feeding of XOS. Overall, this change in the microbiota could be summarized as beneficial, where the abundance of the disease-causing bacteria declined in the treatment groups.

A significant difference in the abundance of bacteria among the treatment groups was observed in this study. Microbial ecology effectively defines the cecal metabolites’ production that affects the physiology of the host [48]. Chondroitin sulfate is one of the glycosaminoglycans influencing the gut barrier defense and microecology [49]. On d 7, the XOS2 group reduced the abundance of genes for chondroitin sulfate degradation I (bacterial) pathway compared to the CON group. A higher turnover of chondroitin sulfate in the cecal lumen happens in pathological conditions of the cecal mucosal surface, e.g., colitis [50]. The pathogenic bacteria utilize the free-form chondroitin sulfate as a source of nutrition and a path to colonize the gut [51], and the metabolites of this pathway aggravate the progress of colitis [52]. A lower abundance of this pathway in the XOS2 chickens suggests the ceca had a less burden of pathogenic bacteria in this group.

Studies suggested that an increase in the MMP of histidine degradation could be microbial biomarkers of fatty liver disease, and prebiotic XOS could alleviate this liver condition [48]. In agreement, we also found that the XOS3 group reduced the MMP of L-histidine degradation I on d 7. Some bacteria are capable of synthesizing thiamine and exchanging this metabolite with other bacteria in gut microbiota, which are incapable of de novo synthesis [53]. The external thiamine-dependent bacteria salvage thiamine and bypass energy demanding de novo biosynthesis of thiamine [54]. On d 21, thiamin salvage pathway II was enriched in the XOS2 and XOS3 groups compared to the CON. Enrichment of the Thiamin salvage pathway and a reduction in histidine degradation have been found protective to colorectal cancer recurrence in human studies [55], and these pathways could be identified as a biomarker for good health in other species, including poultry.

D-xylulose 5-phosphate, produced from D-xylose, can enter into the Calvin-Benson-Bassham cycle to produce energy [56]. An increase in this pathway could be linked to a higher utilization of xylose by the gut microbes. An increase in the pyruvate fermentation to acetate and lactate pathway in the XOS2 group on d 21 indicates the abundance of carbohydrates in the gut, which microbes utilize for acetate and lactate production [57, 58]. The result of our study also showed a higher pyruvate fermentation, which suggests that XOS2 group had utilized a higher amount of carbohydrate in the gut. This excelled fermentation ability might have resulted due to the change in the cecal microbial community in the XOS2 group.

The microbiota's impact extends to cecal SCFA production, while the integrity of the colonic epithelium influences the absorption of free fatty acids (FFAs). FFA receptor genes are expressed in the colonic epithelium and their expression increases the transportation of the FFAs to colonocytes [58, 59]. A compromised epithelial barrier might lead to reduced FFA absorption, increasing their presence in cecal content and vice versa. In this investigation, the XOS2 and XOS3 groups had decreased FFA levels in the cecal digesta of d 21 chickens, indicating increased expression of FFA receptors, increased rate of FFAs uptake to colonocytes, and an enhanced colonic epithelial function [58]. However, no variation in SCFA concentrations among treatments was observed on d 14.

Certain bacteria utilize FFAs, potentially reducing their (FFAs) presence in cecal digesta. Metagenomic analysis indicated that the XOS2 and XOS3 groups decreased the abundance of *Ruminococcus torques* group, which is known for producing SCFAs via fiber fermentation. The decline in SCFAs could also be attributed to reduced *Ruminococcus torques* genus abundance in these groups [60]. Again, metabolites can serve as the substrates for other MMPs. The metabolic pathways showed increased L-isoleucine biosynthesis in the XOS2 and XOS3 groups on d 21. This pathway utilizes propionate to produce L-isoleucine, which could be the reason for the reduction of propionate in cecal digesta in the XOS2 and XOS3 groups [61]. Acetate could participate in cross-feeding gut microbes, debarring the experiment to assess the production in ceca. When monogastric animals consume XOS-enriched diets, XOS (i.e., the building block of some fibers) acts as a prebiotic, shaping the microbiota and fostering the generation of SCFA through gut microbial fermentation [62, 63]. The disparities observed in outcomes between in ovo XOS feeding and dietary XOS feeding experiments could also be attributed to the singular exposure of the subjects to XOS rather than a continuous dietary supplementation.

Besides that reason, fluctuations in SCFA concentration may arise from varying SCFA fluxes to the cecum and the ongoing utilization of SCFAs by MMPs [64]. Therefore, a comprehensive perspective including all bacterial metabolic processes, assessing the cecal digesta for specific metabolites, and the gene expression of respective nutrient transporters in the cecal epithelium is recommended to better understand the effect of XOS on cecal metabolite productions.

## Conclusion

The in ovo feeding of XOS2 and XOS3 modulated the gut microbiota and significantly impacted the metabolic pathways of the microbes inside the gut and SCFA concentration. The in ovo feeding of XOS2 was more effective than the control group in regulating gut microbes and promoting beneficial microbes. However, the reduction of SCFAs in the XOS3 treatment groups did not correspond with the gut microbial ecology and metabolic pathways functioning within the ceca. Future studies could incorporate an evaluation of the cecal nutrient absorption capacity in response to in ovo XOS feeding, which will contribute to establishing a more definitive conclusion on the effect of in ovo XOS on SCFAs production. The MMPs in the treatment groups revealed an uplift in the biosynthesis of thiamin, flavin, amino acids, and carbohydrate utilization in the cecum of the XOS2 and XOS3 group chickens. This prediction of the metabolic pathways could be validated by assessing the metabolite concentration in the cecal digesta in future studies. Overall, our study found that in ovo feeding of XOS2 and XOS3 could beneficially modify gut microbial population and their metabolic pathways.

## Data Availability

The raw sequencing data has been deposited in the NCBI database (Accession number PRJNA1027486).

## Abbreviations

CON: Control
ELISA: Enzyme-linked immunosorbent assay
FFA: Free fatty acid
MMP: Microbial metabolic pathway
PCR: Polymerase chain reaction
SCFA: Short chain fatty acid
XOS: Xylooligosaccharide
XOS2: Xylobiose
XOS3: Xylotriose
